# Treatments for Inhalant and Volatile Substance Misuse: A Scoping Review

**DOI:** 10.7759/cureus.89170

**Published:** 2025-07-31

**Authors:** Gary Lail, Vanessa C Cornelio, Kimberly Go, Kiran Kaur, Piotr Slowik

**Affiliations:** 1 Psychiatry, William Carey University College of Osteopathic Medicine, Hattiesburg, USA; 2 Psychiatry, St. John's Episcopal Hospital, Far Rockaway, USA

**Keywords:** addiction, addiction disorder, addiction psychiatry, inhalant misuse, inhalant use disorder, substance abuse, substance abuse prevention, substance addiction, treatment choices

## Abstract

Inhalant and volatile substance misuse present significant challenges to society, yet the treatment options have largely been overlooked. Currently, there are no FDA-approved pharmacotherapies for inhalant use disorders. Additionally, there are limited nonpharmacological studies focused on the treatment and management of the disorders. These limitations have made it difficult to establish a standardized approach when treating the disorder and have limited the treatment options available to individuals facing the addiction.

This scoping review examines the proposed treatment options for inhalant and volatile substance misuse. It categorizes the proposed treatments and specifically outlines their effectiveness and limiting factors. The review utilized multiple databases such as PubMed, ScienceDirect, and PsycINFO to explore various case reports, public health reports, clinical trials, and community-based programs. There was a focus on studies and reports that specifically explored the pharmacological, psychosocial, supportive, and holistic approaches in treating and managing the disorder.

The supportive approaches primarily focused on medical stabilization during the initial hospitalization. They emphasized the management of the airway, breathing, circulation, disability, and exposure (ABCDE) approach, along with patient decontamination. However, these methods offered limited insight into their long-term effectiveness in addressing inhalant and volatile substance misuse. In contrast, pharmacological approaches did explore long-term outcomes in managing inhalant use disorders. Clinical studies involving medications such as aripiprazole showed significant reductions in sustained inhalant use. Similarly, case reports examining the use of baclofen, naltrexone, and lamotrigine reported varying degrees of success in promoting abstinence from inhalants and volatile substances. Nevertheless, these findings were primarily based on small clinical trials and case reports, thus limiting conclusions about their broader effectiveness. Holistic and multifaceted programs, such as the Youth Solvent Addiction Program (YSAP) and the Mt Theo Program, demonstrated the highest success rates among the interventions reviewed. Many of these residential programs incorporated various culturally specific strategies. While effective in their respective contexts, they may pose challenges for broader replication. Psychosocial interventions, including cognitive behavior therapy (CBT)-based brief interventions and family therapy, also demonstrated a reduction of volatile substance use. Yet, they require larger, more comprehensive studies to better evaluate their efficacy.

The treatment of inhalant and volatile substance misuse has been approached through various mechanisms. Multiple studies and reports involving the supportive, holistic, pharmacological, and psychosocial approaches have successfully demonstrated varying levels of success when managing inhalant and volatile substance misuse. Yet, no FDA-approved pharmacological treatment or standardized approaches have been developed for managing the disorder. The literature review found that many of the approaches have only demonstrated efficacy on smaller scales and were limited by a culturally specific context. Therefore, further research employing large-scale and multifaceted methodologies is necessary to establish the efficacy and replicability of these interventions.

## Introduction and background

As substance use continues to pose significant public health challenges, inhalant and volatile substance misuse has emerged as a concerning yet often overlooked area of study. Inhalant and volatile substance misuse involves the deliberate inhalation of chemical substances to produce psychoactive effects. Wu and Ringwalt (2006) found that 10% of all adults have used an inhalant at least once in their lives [1]. Of the users in the past year, at least 8% found themselves meeting the criteria for inhalant use disorder, which is the abuse and/or dependency on inhalants. These were largely individuals aged 35-49 years with lower education, recent treatment for psychological issues, and co-existing alcohol use disorders [1]. Inhalant and volatile substance misuse can cause significant damage to many organ systems, including pulmonary, cardiac, dermatologic, renal, hematologic, gastrointestinal, hepatic, and neurologic systems [2]. Long-term abuse may also lead to psychiatric, cognitive, and behavioral issues [2]. The misuse may be attributed to the euphoric effects following the direct inhalation of hydrocarbon-based substances [3]. Yet, this mechanism only partly explains why certain hydrocarbon-based inhalants are desirable and may not account for other volatile substances. The type of substance used by patients with inhalant use disorder can vary quite widely, ranging from glues and paints to nail polish remover and cleaning fluids, with the most commonly used inhalants being nitrous oxide and amyl nitrite [1]. This wide range of substances makes it much more difficult to isolate a specific receptor and mechanism of action responsible for the addiction [3]. Thus, researchers are faced with the unique challenge of finding a single treatment option for a disorder influenced by numerous substances.

The wide range of substances, each with distinct mechanisms of action, may be a major barrier to the development of effective and credible treatment options. Therefore, treatment approaches beyond the standard mechanism of action may be required for treating inhalant and volatile substance misuse. Unfortunately, a lack of large-scale research on the treatment of inhalant and volatile substance misuse has left the disorders without a definitive treatment option. While a few systematic reviews have been conducted, they highlight the limited available evidence and the need for further critical analysis. Yet, this has not stopped researchers from successfully attaining positive results from different therapeutic and treatment options. Over the past few decades, researchers have studied various therapeutic approaches for the treatment and management of inhalant use disorders. Firstly, supportive approaches have been evaluated for the safety and recovery of an acutely ill patient. Various pharmacological approaches have also been studied with varying levels of efficacy. Holistic and multifaceted programs, such as residential programs, have also demonstrated promising results in the management and treatment of the disorders. Finally, psychosocial approaches, such as cognitive behavior therapy (CBT) and family therapy, have also been tested to manage inhalant use disorders. This scoping review aims to provide a critical analysis and to discuss the range of treatment approaches. Furthermore, it seeks to provide novel suggestions and potential modifications to guide the development of a definitive treatment option for inhalant and volatile substance misuse.

## Review

Methods

Protocol

This scoping review was conducted using the five-stage framework described by Arksey and O’Malley, which includes (1) formulating the review question; (2) conducting a comprehensive search for relevant studies; (3) selecting appropriate studies based on predefined inclusion and exclusion criteria; (4) identifying relevant data; and (5) gathering, summarizing, and presenting the findings [4].

Eligibility Criteria

To be included in this scoping review, studies need to address clinical treatments for inhalant and volatile substance misuse. Articles were considered eligible if they were peer-reviewed, written in English, included human participants, published between 1982 and 2023, and evaluated a treatment targeting inhalant and volatile substance misuse. Quantitative, qualitative, and mixed-method studies were included to ensure comprehensive coverage of current knowledge. Articles were excluded if they were not peer-reviewed or did not discuss specific treatments targeting inhalant and volatile substance misuse.

Information Sources

To identify relevant studies, the following databases were searched: PubMed, ScienceDirect, and PsycINFO. The authors of this review did initial data collection on November 11, 2024. The titles and abstracts of the articles were evaluated for significance for this scoping review and if they met the inclusion and exclusion criteria. After careful assessment, 18 articles were used for this scoping review.

Search Strategy

The search strategy was designed to target the research question: “What treatments have been proposed for inhalant and volatile substance misuse?” Database searches use a combination of search terms, synonyms, and Boolean operators to ensure a comprehensive search for relevant studies. Initial search terms included “inhalant,” “volatile substance,” and “inhalant use disorder,” along with “misuse,” “abuse,” and “dependence,” in combination with terms such as “treatment,” “prevention,” and “intervention.”

Selection of Sources of Evidence

**Figure 1:**
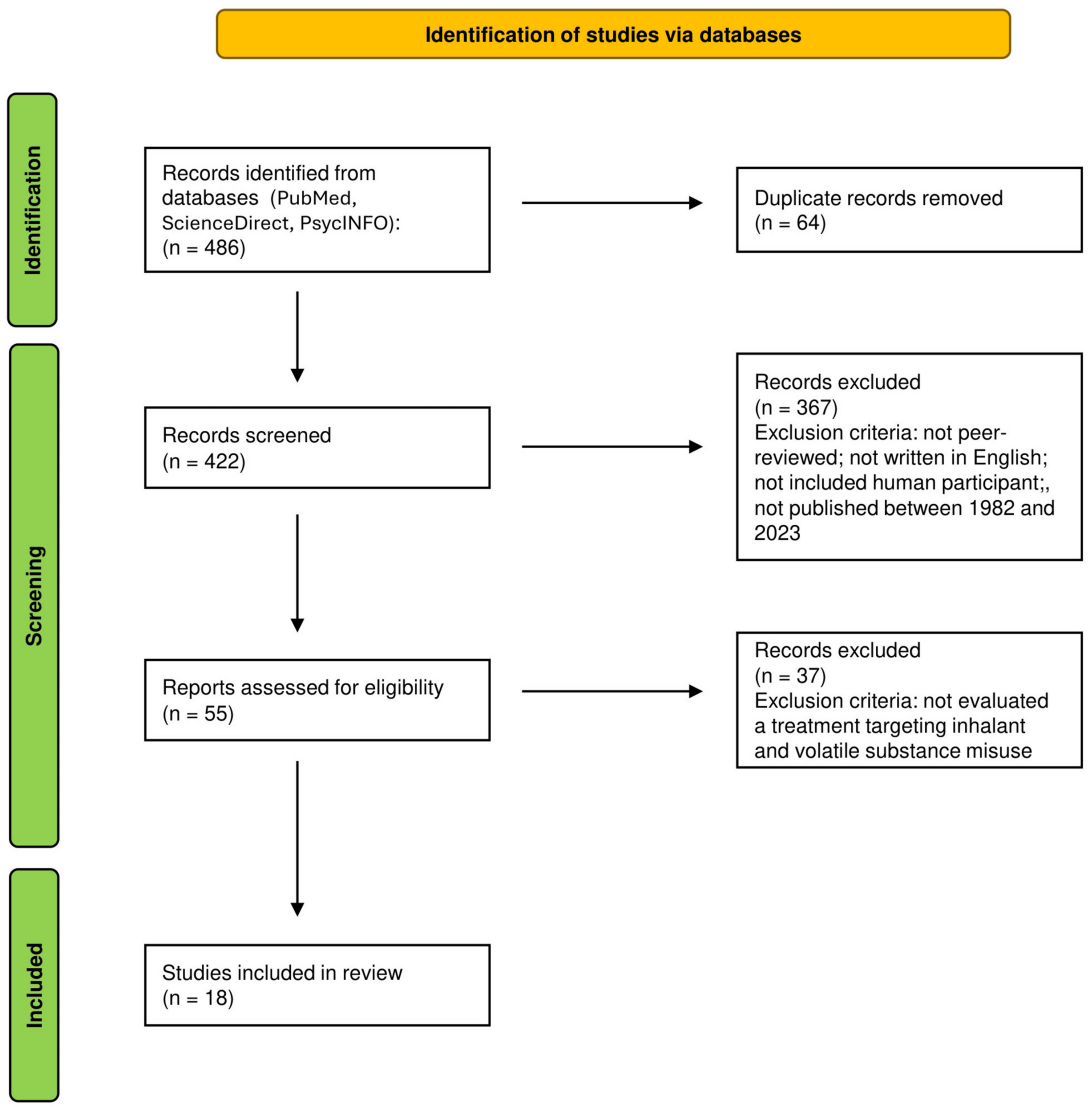
A PRISMA flow diagram of the study identification, screening, and selection process PRISMA: Preferred Reporting Items for Systematic Reviews and Meta-Analyses

The authors collectively examined the eligibility criteria, along with the results of the database searches, to ensure the selection of appropriate studies. The titles and abstracts of the articles were evaluated for their importance in this scoping review and to determine if they met the eligibility criteria. Any disagreements regarding article selection were resolved through thoughtful discussion among the authors. This study's identification, screening, and selection process is depicted by a Preferred Reporting Items for Systematic Reviews and Meta-Analyses (PRISMA) flow diagram (Figure 1).

Data Charting Process

The data charting process was done using Microsoft Excel (Microsoft Corp., Redmond, WA). The authors identified relevant data from the selected articles and entered the information into an Excel form. The most relevant data to include in the scoping review was determined through meticulous evaluation and agreement among the authors.

Data Items

The relevant data that were extracted from the selected articles discussed proposed clinical strategies for treating and managing inhalant and volatile substance misuse. The data were included if they helped the scoping review summarize the current knowledge on potential treatment approaches.

Synthesis of the Results

The studies discussed in this scoping review were categorized into distinct therapeutic approaches for the treatment and management of inhalant use disorders: supportive, pharmacological, holistic, and psychosocial approaches.

Results and discussion

Supportive Approach

Evidence: The supportive treatment of inhalant and volatile substance toxicities aims to medically stabilize the patients upon initial hospitalization. There is currently no FDA-approved treatment for Inhalant Use Disorder; thus, this approach entails a variety of measures to help stabilize and minimize toxicity to the patient [3]. Firstly, monitoring and addressing the patients through the airway, breathing, circulation, disability, and exposure (ABCDE) approach is essential to ensure the safety and recovery of an acutely ill patient [5]. The approach is widely used throughout the United States and has been commonly used as the first line of defense when approaching individuals with inhalant toxicities.

In addition to the ABCDE approach, there are a variety of supportive methods used to counteract the most common physiological side effects of inhalants. For example, many of the volatile substances used by these patients have been found to be pro-arrhythmic. Therefore, continuous ECG monitoring is essential in identifying any cardiac changes [2]. Another common side effect of inhalant use is myocardial irritation. Thus, catecholamines should be used cautiously and potentially replaced with certain beta (β)-blockers due to their cardioprotective nature [6]. Hypotension can also be a commonly presenting symptom in inhalant toxicity. Therefore, low blood pressure can be supportively treated with fluids and positional placement. During these toxicities, patients can also develop varying levels of agitation and seizure activity. To help manage these patients and maintain patient safety, benzodiazepines can be used for management [7]. Additionally, decontamination of the patient's clothing and skin may be essential to prevent further toxicities, burns, or irritation [8].

Limitations: In summary, a wide range of supportive measures are utilized as first-line treatments to help medically stabilize the patient. However, these are only basic measures to help stabilize a patient and are not the “definitive treatment” in treating the disorder. We suggest that further research must be conducted to help develop an effective protocol when approaching a patient with inhalant use toxicity. Due to the lack of FDA-approved treatments, a well-researched and evidence-based protocol may be an extremely valuable tool when approaching these hospitalizations.

Pharmacological Approach

Evidence: Pharmacological treatments for inhalant use disorder have been tested and experimented with for years. Despite the varying levels of success, there has been no FDA-approved medication for the disorder, and studies continue to prove their efficacy and validity. Yet, there is a wide range of medications that have been proposed, ranging from antipsychotics to antiepileptics. This range indicates that multiple mechanisms may need to be targeted when approaching the disorder. 

Beginning with the antipsychotics, aripiprazole is among the first medications to demonstrate efficacy when treating inhalant use disorder. A study was conducted using seven adolescents meeting the Diagnostic and Statistical Manual of Mental Disorders, Fifth Edition (DSM-5) criteria for inhalant use disorder. They were placed on an average dose of 11 mg per day for six months. In the treatment period of 3 months, the study found that 57.1% of the participants remained fully abstinent from inhalant use [9]. The remaining 42.9% displayed a significant reduction in inhalant use following six months of treatment (p=0.01) [9]. One key feature to consider in this study is that all of the participants were also diagnosed with conduct disorder according to the DSM-5 criteria. Therefore, this study exemplifies how aripiprazole may be an effective treatment option when treating patients with both disorders, but does not illustrate whether this treatment will work on individuals only diagnosed with inhalant use disorder.

Haloperidol and carbamazepine have been studied in the context of inhalant use disorder, but focused mainly on the psychotic symptoms associated with the disorder. Therefore, the study chose to focus on treating specifically inhalant-induced psychotic disorder. The study assessed 40 male patients diagnosed with both inhalant dependence and inhalant-induced organic mental syndrome and randomly assigned them to either haloperidol or carbamazepine. The study found a 48.3% reduction in the carbamazepine group and 52.7% in the haloperidol group (p<0.001) [10]. Thus, the study successfully demonstrated a significant reduction in psychotic symptoms in both treatment groups. It is important to note that the study was evaluating the psychotic symptoms, but it does not specifically address the reduction in the use of inhalants. Therefore, the study may provide useful information on managing the disorder, but it does not give us much information on how to treat the disorder itself.

There have been multiple other pharmacological therapies proposed throughout the last few decades, but many are supported by case reports. Baclofen has been a strong candidate, providing much preliminary support as a potential treatment option. A case report of a 14-year-old with inhalant dependence syndrome demonstrated complete abstinence from inhalant use while using baclofen during a 10-month period [11]. Similarly, a 2008 case series studied three adolescent male patients with inhalant dependence and treated them with baclofen. The study found that all participants reported a significant decrease in withdrawal symptoms within 48 hours of continuing the medication and were asymptomatic during the hospitalizations [12]. Other medications have also found similar preliminary data supporting reduced inhalant use. For example, a 41-year-old male patient misusing nitrous oxide had decreased use during a one-month period of naltrexone treatment [13]. In another case, a 21-year-old male patient diagnosed with inhalant dependence reported complete abstinence from inhalants following a six-month period of treatment with lamotrigine [14]. While the case reports and the smaller studies demonstrate varying levels of success, they provide only limited evidence to support these interventions as a viable therapeutic option.

Limitations: While the pharmacological therapies and treatments have shown promising data, the results are limited by their small sample sizes. The studies on aripiprazole, haloperidol, and carbamazepine have demonstrated success, yet large-scale studies are required to determine their efficacy. Nevertheless, these smaller-scale studies are critical to understanding the mechanisms revolving around inhalant and volatile substance misuse. They provide evidence that dopaminergic or neuronal excitability may hold a key role in the stimulating and addictive nature of certain inhalants. Therefore, future research specifically targeting dopaminergic receptor blockade and reducing neuronal excitability may provide therapeutic evidence for these alternative treatment options.

The case reports on baclofen, naltrexone, and lamotrigine may indicate success on an individual basis. Yet, the individual's response to treatment cannot be generalized to a broader population. Therefore, larger-scale research into these pharmacological treatments needs to be conducted to prove their efficacy. Furthermore, we are unable to establish causality without further evaluating for confounding variables or underlying conditions. Therefore, we suggest conducting controlled research, as this will better demonstrate their therapeutic capabilities and establish a more causal relationship. Research into these novel approaches is critical because they may suggest overlooked mechanisms contributing to inhalant misuse. Therefore, a careful evaluation of these case reports is essential to refining and developing new therapeutic approaches.

Proposed pharmacological treatments were summarized in a table format based on the medication, dosage, sample size, and findings (Table 1).

**Table 1:** Summary of the proposed pharmacological treatments for inhalant use disorder

Pharmacological approach	Dosage	Sample size	Findings	Study
Aripiprazole	11 mg per day for six months	n=7	57.1% of participants remained fully abstinent from inhalant use, remaining 42.9% displayed a significant reduction in inhalant use	Erdogan and Yurteri (2010) [9]
Haloperidol	5 mg, three times a day for five weeks	n=20	52.7% reduction in psychotic symptoms	Hernandez-Avila et al. (1998) [10]
Carbamazepine	200 mg, three times a day for five weeks	n=20	48.3% reduction in psychotic symptoms	Hernandez-Avila et al. (1998) [10]
Baclofen	40 mg per day for 10 months	n=1	Complete abstinence from inhalant use	Kandasamy et al. (2015) [11]
Baclofen	50 mg per day during a period of hospitalization	n=3	Significant decrease in withdrawal symptoms within 48 hours	Muralidharan et al. (2008) [12]
Naltrexone	100 mg per day for one month	n=1	Decreased use of nitrous oxide	Ickowicz et al. (2020) [13]
Lamotrigine	100 mg per day for six months	n=1	Complete abstinence from inhalant use	Shen (2007) [14]

Holistic Approach

Evidence: A holistic and multifaceted approach is distinct because it simultaneously tackles multiple psychosocial and environmental factors contributing to the inhalant and volatile substance misuse. This method may be more sophisticated than a simple pharmacological approach, yet its complexity addresses various underlying factors that contribute to the pathological process of the disorder. A few Canadian and Australian programs have utilized this approach and have demonstrated promising results by addressing the psychological, social, and environmental elements potentially contributing to the inhalant and volatile substance misuse.

Residential treatment centers in Canada were among the first to successfully utilize this holistic approach when treating individuals suffering from inhalant misuse. These centers created the National Native Youth Solvent Addiction Program (NNYSA), which utilized both Western and First Nation techniques, creating the Residential Model. This model aimed to “strengthen their inner spirit” by focusing on the patient’s resilience when addressing inhalant misuse. The program successfully found that in 2000 and 2001, respectively, 82% and 95% of clients reported six-month abstinence from inhalants following the treatment [15]. This data is quite promising, but unfortunately, the completion rate of these programs has been variable, ranging as low as 11% [16]. Therefore, the high rates of abstinence may be contingent on full completion of the program. The variable completion rates may indicate an underlying challenge with the complexity of the approach. Furthermore, it may suggest that program refinement, focusing more on participant retention, may be critical. A root-cause analysis and program model adjustments may be required to address these varying completion rates and to help develop a more promising therapeutic option.

The Youth Residential Solvent Treatment Program (YSAP) was another residential program based in Canada. It similarly utilized both Indigenous and Western practices to tackle Volatile Substance Misuse (VSM). The study evaluated patients who received YSAP treatment between 2007 and 2009. The study found that 90 days following the YSAP program, 50% of youth reported abstinence from volatile substances [17]. Additionally, 51% of these youth also reported having no urge to misuse volatile substances [17]. Interestingly, 180 days following the YSAP treatment, these numbers increased to 74% of youth reporting abstinence and 68% reporting no urge to misuse [17]. Due to high client mobility, only 154 individuals were included in these statistics, which only reflects 40% of the total individuals who participated in the program. Therefore, a large portion of individuals who participated in the program are being withheld from these vital statistics, which introduces a unique challenge when evaluating the program's efficacy.

The Mt Theo program, based in Australia, is another interesting program that evaluated inhalant misuse. The program was essential to curb the addiction in the Yuendemu Aboriginal community, as it was struggling with a petrol-sniffing crisis. The Mt Theo program utilized a holistic approach by engaging in cultural activities, hunting, discussions with elders, and more. The program's multifaceted approach helped the community successfully tackle the crisis while demonstrating impressive efficacy. Over a nine-year period of the Mt Theo Program, it outstandingly reduced the number of petrol sniffers from 70 to 0 [18]. The high treatment rate demonstrates that a multifaceted approach may be critical to treating inhalant misuse. Interestingly, the program required users to relocate 50 km away from the nearest major road. Further evaluation of the program's isolation is necessary to better understand its impact on treatment outcomes and to determine whether these outcomes are influenced by isolation itself, program-specific factors, or a combination of both.

Limitations: The methodology utilized by these programs may be regarded as culturally specific. A culturally specific program may be difficult to replicate on a larger scale due to various intricacies. For example, accurate implementation, culturally specific norms, and underlying societal differences may present challenges if the culture is not fully understood. Therefore, identifying the key components that contributed to the program's success may be essential for effectively replicating the model. Regardless of these potential limitations, these programs demonstrate how holistic medicine and multifaceted approaches can effectively address the psychosocial and environmental challenges behind inhalant and volatile substance misuse.

Psychosocial Approach

Evidence: The psychosocial approaches are among the most commonly used treatments for inhalant use disorder. Psychosocial techniques such as CBT-based brief intervention and family therapy are frequently used for managing inhalant and volatile substance misuse and related disorders. Unfortunately, limited studies have evaluated their efficacy when specifically treating inhalant use disorders. Therefore, their therapeutic use may be attributed to their efficacy demonstrated with other substance use disorders.

CBT-based brief intervention is commonly used to help treat patients suffering from VSM dependency. According to Ogel and Coskun (2011), CBT-based brief intervention can successfully reduce VSM in adolescents [19]. In their study, 62 adolescent male patients who were clinically diagnosed with “volatile substance dependence” were divided equally into two groups. The experimental group consisted of a CBT-based brief intervention plus an educational program, while the control group utilized just the educational program. The study found that after one year, 38.2% of the experimental group and 58.1% of the control group reported continuing VSM in the past three months [19]. These results demonstrated a significant decline in VSM during the one-year follow-up. Although the study demonstrates a significant reduction in VSM following a CBT-based brief intervention, the small sample size limits the power of the study. Therefore, a larger sample size is required to help illustrate the efficacy of this treatment option. Furthermore, we are unable to determine the isolated effect of CBT, as both groups were exposed to an educational program. Therefore, refining the study to isolate the effect of CBT-based brief intervention may be critical to determine whether CBT alone is a viable therapeutic option.

Another commonly used psychosocial approach is family therapy. This model addresses the familial aspects, such as boundary setting, enhancing communication, and reframing family roles. Framrose (1982) studied 35 families dealing with solvent misuse and placed them into a family therapy program [20]. At a six-month follow-up, 74% of those families experienced a “good outcome,” which was characterized by cessation of solvent misuse and improved family functioning [20]. This study demonstrates the value of family support and illustrates how family therapy can reduce solvent misuse while simultaneously improving overall family function. This study also utilizes a small sample size. Therefore, a larger sample size will help evaluate the efficacy of family therapy in reducing solvent misuse. Yet, this study demonstrates a key social factor in inhalant and solvent misuse. It illustrates how substance misuse may go beyond the individual level and explores the more dynamic nature of substance use disorders.

## Conclusions

The different treatment approaches have demonstrated varying levels of success when treating and managing inhalant-related disorders. Yet, the majority of treatments have only been tested on a small sample size or studied within a case study. Thus, providing only limited information on the efficacy of the treatment on a larger scale. Furthermore, many of the treatment options may be difficult to replicate due to cultural specificity and community relevance. Yet, these limitations can be overcome through additional research, study modifications, and exploring alternatives.

There are also many benefits to having a large range of preliminary data. Firstly, the large variety of treatment options can help guide large-scale research and development. These studies have demonstrated success on these smaller scales, which is essential before conducting experiments with a larger group of patients. Furthermore, a wide collection of preliminary data can help determine whether treatment options should be approached from a more pharmacological, psychosocial, or holistic standpoint. Furthermore, potentially using multiple therapeutic approaches simultaneously may be the most effective treatment protocol. Therefore, through further research and development, a definitive treatment option can be developed to help manage individuals suffering from inhalant and volatile substance misuse.
